# Two combinations of house dust mite allergens show similar performance than extracts for asthma diagnosis

**DOI:** 10.3389/falgy.2026.1816013

**Published:** 2026-04-10

**Authors:** Victoria Marrugo, Josefina Zakzuk, Randy Reina, Karen Donado, Ronald Regino, Isabel Gil, Caterine Meza, Dilia Mercado, Enrique Fernández-Caldas, Leonardo Puerta, Nathalie Acevedo, Luis Caraballo

**Affiliations:** 1 Institute for Immunological Research, University of Cartagena, Cartagena de Indias, Colombia; 2 Respiratory and Allergy Clinic, Cartagena de Indias, Colombia; 3 University of South Florida, Tampa, FL, United States

**Keywords:** *Blomia tropicalis*, *Dermatophagoides pteronyssinus*, diagnostic performance, FeNO, house dust mite, molecular allergy diagnosis, recombinant allergens

## Abstract

**Introduction:**

Molecular allergy diagnostics for house dust mite (HDM) sensitization includes both allergenic molecules and extracts, but extracts have batch-to-batch variability, incomplete allergen representation and cross-reactivity, which confound results and reduces test accuracy. However, although using extract-free arrays could solve these problems, there is no study that formally supports this change. Therefore, the aim of this study was to compare the diagnostic performance and clinical relevance of measuring specific IgE to *Dermatophagoides pteronyssinus* and *Blomia tropicalis* extracts vs. two allergen combinations, Blo t 2/Blo t 5/Blo t 21 and Der p 1/Der p 2/Der p 23.

**Methods:**

In 201 adults with asthma and matched controls, diagnostic performance of specific IgE towards molecular allergen combinations and extracts were compared using receiver operator characteristics analysis, with physician-diagnosed asthma as reference standard. Associations between specific IgE (extracts and combinations) and type 2 inflammation biomarkers (fractional exhaled nitric oxide and blood eosinophils) were also evaluated.

**Results:**

Specific IgE frequencies and levels were higher in patients. Allergen combinations and extracts showed equivalent performance. The area under the curve of the combination Blo t 2/Blo t 5/Blo t 21 was similar to that of the *B. tropicalis* extract: 0.783 and 0.808 respectively (*p* = 0.42). Likewise, the area under the curve of the combination Der p 1/Der p 2/Der p 23 was 0.793 and that of extract was 0.788 (*p* = 0.8). Notably, IgE response to *D. pteronyssinus* allergens was more specific than the extract and significantly associated with fractional exhaled nitric oxide and blood eosinophils.

**Conclusion:**

Our findings provide, for the first time, direct evidence that specific allergen combinations have similar diagnostic performance to HDM extracts in molecular diagnostics, improving test accuracy and supporting a shift toward standardized, molecular-resolved diagnostic strategies. In addition, we found that allergen combinations, and not HDM extracts, are strongly associated with type 2 inflammation biomarkers, supporting their use for personalized asthma management.

## 1 Introduction

House dust mites (HDM), particularly *Blomia tropicalis* and *Dermatophagoides pteronyssinus,* are major indoor allergens and key contributors to allergic diseases such as asthma and rhinitis (1, 2). Although HDM extracts are still commonly used in molecular allergy diagnostics (MAD) platforms, they have significant limitations, including batch-to-batch variability, incomplete representation of allergenic proteins and the presence of cross-reactive components (3–6), which can hinder genuine sensitization profiles and complicate personalized management (2, 7).

MAD, also known as Component Resolved Diagnosis (CRD), have emerged as transformative approaches that use recombinant allergens to precisely map IgE reactivity (2, 8). This strategy enhances diagnostic accuracy and informs targeted immunotherapy. However, there is a lack of direct comparisons between the diagnostic performance of HDM extracts and defined allergen panels. This knowledge gap is especially important in tropical regions, where individuals are often exposed to multiple HDM species and helminth parasites (9).

For HDM allergy, MAD platforms still typically include extracts, but there is growing interest in replacing them with defined combinations of key allergens. For *D. pteronyssinus*, Der p 1, Der p 2 and Der p 23 are well characterized and have demonstrated high clinical relevance (10–17). Similarly, for *B. tropicalis*, Blo t 2, Blo t 5 and Blo t 21 have been identified as the most clinically relevant sensitizers (18–20).

In this study, we directly compare, for the first time, the asthma diagnostic performance and clinical impact of HDM extracts vs. defined allergen combinations. Our goal is to determine whether extracts can be effectively omitted from MAD platforms. By rigorously evaluating the clinical value of specific allergen panels, we aim to provide evidence supporting their integration into routine diagnostic and management practices.

## 2 Materials and methods

### 2.1 Study design and population

This was an observational, cross-sectional case–control study conducted in Cartagena, Colombia. Adult patients with asthma were recruited from three specialized centers in this city: The Respiratory and Allergy Clinic, The RESPIRA program (MutualSER at Caminos IPS) and the Allergy Service of the Institute for Immunological Research at the University of Cartagena. Recruitment was conducted as part of the EPAT project “Studies on the pathogenesis of asthma in the tropics: opportunities for knowledge generation and innovation in biomedicine” (EPAT project, BPIN 2020000100405) between February 2023 and June 2025.

Asthma was diagnosed according to GINA 2024 guidelines (21). Asthma patients underwent a detailed clinical evaluation, including medical history, complete blood count, skin prick testing (SPT), spirometry, and fractional exhaled nitric oxide (FeNO). Control subjects were confirmed to have no asthma or other allergic diseases (allergic rhinitis, atopic dermatitis). Individuals with a history of autoimmunity, immunosuppression, or active neoplasia were excluded. All participants provided written informed consent. The study was approved by the Ethics Committee of the University of Cartagena (Minute No. 128, November 14, 2019).

### 2.2 Sample size

Sample size was estimated using the Cleveland Clinic Sample Size Calculator for Receiver Operating Characteristic (ROC) analysis. Assuming a minimum area under curve (AUC) of 0.70, a statistical power of 90% and a two-sided alpha error of 0.008 (corrected by multiple testing), the minimum required size was 132 individuals (66 cases and 66 controls).

### 2.3 Skin prick test

SPTs were performed with standardized extracts from Inmunoteck S.L. (Alcalá de Henares, Madrid, Spain) for *D. pteronyssinus*, *D. farinae*, *B. tropicalis*, dog epithelium, cat epithelium, grass mix, *Periplaneta americana* and *Aspergillus fumigatus*. Histamine and 50% glycerol served as positive and negative controls, respectively. A drop of each allergen was placed on the skin and a prick was made with a sterile lancet (PrickLan®, Sumivitales, Colombia). Wheal and erythema sizes were measured in millimeters after 15 min. A positive result was defined as a wheal diameter (WD) 3 mm greater than the negative control.

### 2.4 Molecular allergy testing

Blood samples were collected in EDTA-coated tubes from 2023 to 2025. Plasma was separated by centrifugation at 1,500 rpm for 15 min at 4 °C and stored at −80 °C until analysis.

Specific IgE was measured by an indirect ELISA, as previously described (22–25), using a panel of individual allergens and extracts for *B. tropicalis*, *D. pteronyssinus* and *Ascaris lumbricoides*. The panel included six *B. tropicalis* allergens (Blo t 2, Blo t 5, Blo t 10, Blo t 12, Blo t 13, and Blo t 21); three *D. pteronyssinus* allergens (Der p 1, Der p 2, and Der p 23); and two *A. lumbricoides* allergens (ABA-1 and Asc l 5).

Briefly, allergens and extracts were diluted in carbonate/bicarbonate buffer and coated onto 96-well microtiter plates (Immulon® 4 HBX, Thermo, US) overnight at 4 °C (0.5 µg/well for individual allergens, 5 µg/well for extracts, except ABA-1 and *A. lumbricoides* extract at 1 µg/well). Plates were washed (PBS 0.1%-Tween 20) and blocked with PBS containing 1%-BSA and 0.02% sodium azide for three hours at room temperature (RT).

After washing, serum samples (diluted 1:5 in blocking buffer) were added in duplicate and incubated overnight at RT. Plates were washed and incubated for two hours at RT with alkaline phosphatase-conjugated anti-human IgE antibody (Sigma-Aldrich, US Cat. A3525; 1:2000 dilution). Following a final wash, p-nitrophenyl phosphate substrate (Sigma-Aldrich, US Cat. N2640) was added. Optical density (OD) was measured at 405 nm after a 30 min incubation in the dark using Multiskan GO spectrophotometer (Thermo Scientific, US). A serum negative for all allergens and extracts and a serum positive for *B. tropicalis* extract (diluted from 1:5 to 1:320) were included in each run as inter-assay reproducibility controls. Intra-assay coefficient of variation (CV) of less than 10% and an inter-assay CV of less than 20% were obtained. Additionally, blocking buffer without serum was added in duplicate as a blank in each assay. Samples were considered positive when the OD exceeded the defined cut-off values.

### 2.5 Production of recombinant allergens

With the exception of native Der p 1 (Inbio, US. Cat. NA-DP1-1), all recombinant allergens were produced at the Institute for Immunological Research. Sequences for Blo t 2, Blo t 10, Blo t 13, Der p 2, and Der p 23 were obtained from GenBank; sequences for Blo t 5, Blo t 12, Blo t 21, ABA-1, and Asc l 5 were derived from cDNA libraries. Sequences were synthesized and cloned into expression vectors: pET45b + (Blo t 2, Blo t 10, Blo t 12, Blo t 13, Asc l 5, Der p 2 and Der p 23); pET100 (Blo t 5 and Blo t 21), and pGEX-1*λ*T (ABA-1). Proteins were expressed in either E. coli Origami or BL21 DE3 strains. Purification protocols followed previous published methods for each allergen (18, 19, 26–32). Protein sequences and accession codes are presented in Supplementary Table 1.

### 2.6 Preparation of allergen extracts

*B. tropicalis* extract was obtained from locally collected and cultured mites as previously described (33–35). *A. lumbricoides* extract was prepared from adult worms obtained from an infected patient in Cartagena (22, 32). The *D. pteronyssinus* extract was commercially sourced (Inmunotek S.L. Alcalá de Henares, Madrid, Spain).

### 2.7 Statistical analysis

From an initial sample of 341 asthma patients and 339 healthy controls, propensity score matching (PSM) was applied to balance cases and controls by age and sex, resulting in 201 matched case–control pairs. Balance was assessed using standardized mean differences (SMD). Data are presented as means with standard deviations (SD) or medians with interquartile ranges (IQR). ROC curves were generated using predicted probabilities from logistic regression models with log-transformed allergen-specific IgE levels as predictors and asthma status as the outcome. The area under the curve AUC was calculated for each allergen. Multivariable logistic regression models were fitted using selected combinations of allergen, with combinations informed by a preliminary random forest analysis (Supplementary Figure S1).

Optimal OD cut-off values for sensitization were determined using Youden's index (Supplementary Table S2). Sensitization to HDMs or specific allergen panels was defined as positivity to at least one component in the respective battery. Sensitivity, specificity, positive predictive value (PPV), and negative predictive value (NPV) were calculated for individual allergens and their combinations. The association between allergen sensitization and asthma was estimated using conditional logistic regression, with results expressed as odds ratios (OR) and 95% confidence intervals (CI). Correlations between SPT results and specific IgE levels were assessed using Spearman's rank correlation coefficient and the Phi (*φ*) coefficient. Differences in FeNO levels (parts per billion) and blood eosinophil counts (cells/µL) between sensitized and non-sensitized asthma patients were analyzed with Mann–Whitney tests. Generalized linear models (GLMs), adjusted for age and sex, were used to evaluate associations between FeNO or eosinophil counts and specific IgE sensitization status. Agreement between sensitization to individual allergens, allergen combinations, and allergenic extracts was assessed using Cohen's Kappa coefficient. Both asthma patients and controls were included in the analysis. Statistical analysis was performed using SPSS version 25 (IBM Corporation, New York, USA).

## 3 Results

### 3.1 Differences of sensitization between asthma cases and controls

The sociodemographic and clinical characteristics of the study population are summarized in Tables 1, 2, respectively. Most asthma patients were allergic to HDM, as determined by both by SPT and MAD. The frequency of sensitization was 73.0% for either HDM extract and 70.6% for any of the defined allergen combinations. Based on GINA 2024 criteria (21), asthma severity was classified as mild in 60.7% of patients and moderate in 38.8%.

**Table 1:** Demographic characteristics of the study population.

Demographics	Asthma patients (*n* = 201)	Controls (*n* = 201)	*P* value
Age (years), Median (IQR)	35 (24–50)	33 (23–47)	0.3
Females, *n*. (%)	139 (69.2)	138 (68.6)	1.0
BMI (kg/m^2^), Median (IQR), *n*.	26 (23–30), 183	26 (23–29), 183	0.5
Socioeconomic characteristics			
Completed technical- University education, *n*. (%)	114/186 (61.3)	120/186 (64.5)	0.4
Urban area of residency, *n*. (%)	183/189 (96.8)	186/189 (98.4)	0.5
Low socioeconomic status (1–2), *n*. (%)	150/189 (79.3)	141/189 (74.6)	0.3
Access to sewer service, *n*. (%)	160/179 (89.4)	169/179 (94.4)	0.1
Access to clean water service, *n*. (%)	172/179 (96.1)	172/179 (96.1)	1.0
Access to garbage collection service, *n*. (%)	175/179 (97.8)	177/179 (98.9)	0.6
Access to natural gas service, *n*. (%)	177/178 (99.4)	173/178 (97.2)	0.2

**Table 2:** Clinical characteristics of asthma patients (*n* = 201).

Clinical characteristics	Frequency
History of allergic comorbidities	
Allergic rhinitis, *n*. (%)	170/201 (84.6)
Skin Prick Test results	
Sensitization to *B. tropicalis* extract, *n*. (%)	107/178 (60.1)
Sensitization to *D. pteronyssinus* extract, *n*. (%)	94/178 (52.8)
Sensitization to HDM extracts (Bt and/or Dp), *n*. (%)	130/178 (73.0)
Positive ELISA results	
IgE to *B. tropicalis* allergens combination (Blo t 2/Blo t 5/Blo t 21), *n*. (%)	121/201 (60.2)
IgE to *D. pteronyssinus* allergens combination (Der p 1/Der p 2/Der p 23), *n*. (%)	118/201 (58.7)
IgE to HDM (Bt and/or Dp allergen combinations), *n*. (%)	142/201 (70.6)
Biomarkers	
FeNO (ppb), Median (IQR), *n*.	47 (25–80) 173
Blood eosinophils (cells/mm^3^), Median (IQR), *n*.	240 (140–390) 169
Asthma severity	
Mild asthma, *n*. (%)	122/201 (60.7)
Moderate asthma, *n*. (%)	78/201 (38.8)
Severe asthma, *n*. (%)	1/201 (0.5)
Lung function tests	
FEV_1_ (% Predicted), Median IQR, n.	79 (68–89) 187
FVC (% Predicted), Median IQR, n.	90 (83–99) 187
FEV_1_/FVC, Median IQR, n.	0.74 (0.64–0.81) 187

**Figure 1:**
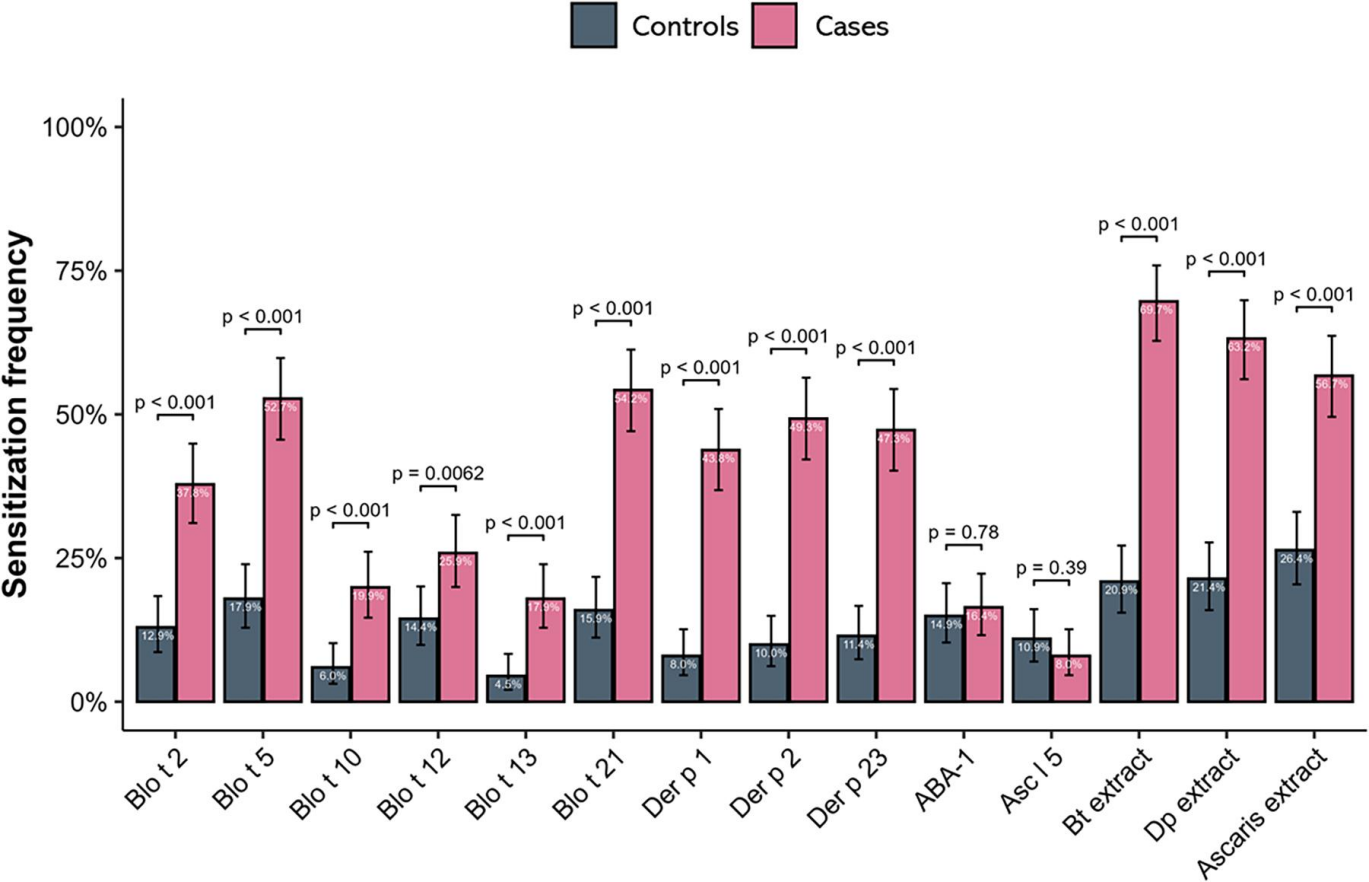
Ige sensitization frequency of asthma patients (cases) and controls to allergens and extracts. IgE levels were measured by indirect ELISA (average of duplicate variations < 10%), using individual cutoff values to determine positivity for each allergen and extract. Logistic regression was used to calculate the difference between groups, with a confidence interval of 95%. Percentages, error bars and *p*-values are shown. Bt extract, *B. tropicalis* extract; Dp extract, *D. pteronyssinus* extract; Al extract, *A. lumbricoides* extract.

**Figure 2:**
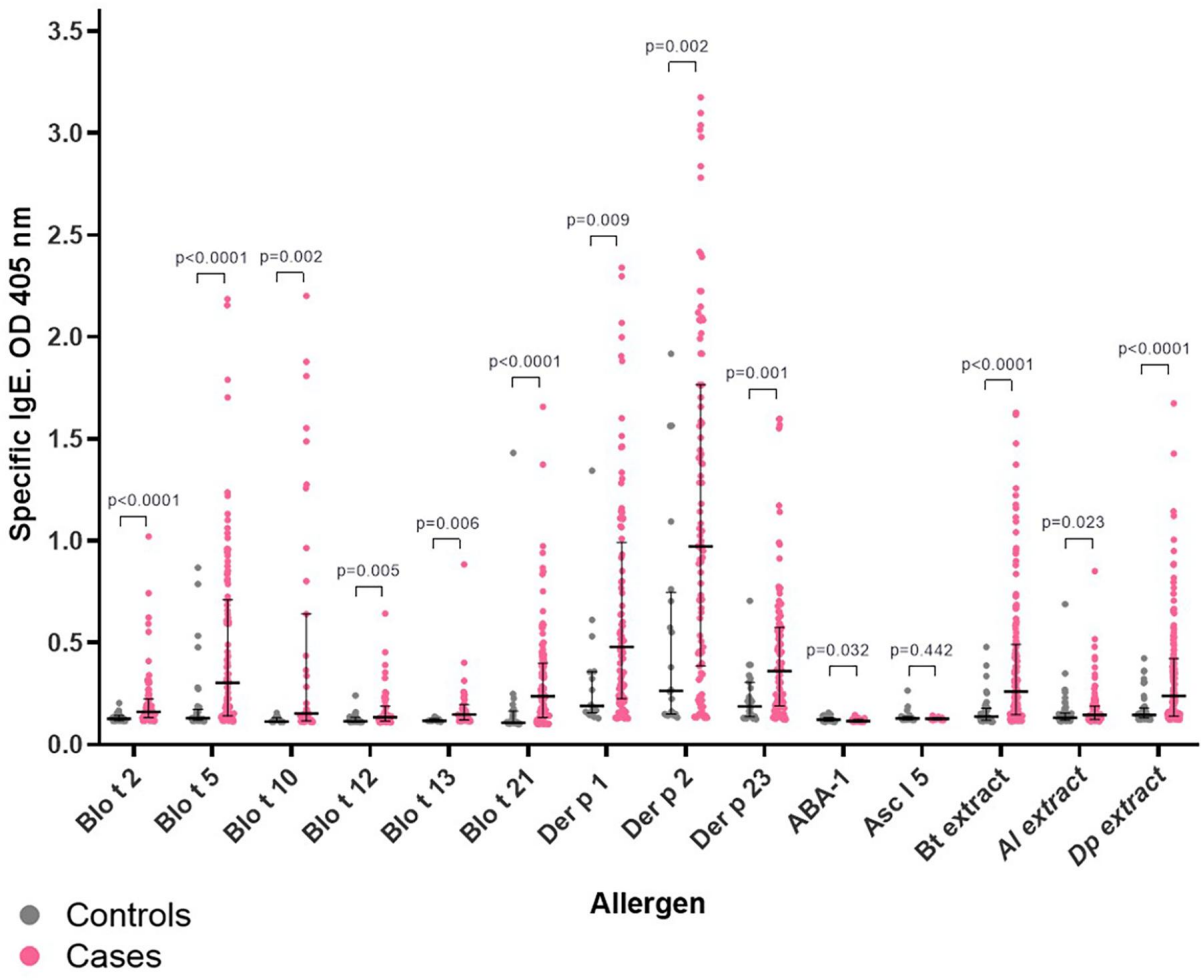
Specific IgE levels of asthma patients (cases) and controls. Optical density (OD) at 405 nm was obtained by indirect ELISA and positive OD levels of asthma patients and controls were used to compare both groups. The difference between median of IgE levels was calculated using the Mann–Whitney U test for non-parametric data. The *p*-value was calculated using a confidence Interval of 95%. Bt extract, *B. tropicalis* extract; Dp extract, *D. pteronyssinus* extract; Al extract, *A. lumbricoides* extract.

As expected, sensitization to HDM extracts and to several individual allergens was significantly more frequent in asthma patients than in controls (Figure 1, Table 3). Specific IgE levels were also higher in asthma patients (Figure 2, Supplementary Table S3). In contrast, there were no significant differences in IgE reactivity to the Ascaris-specific markers ABA-1 and Asc l 5.

**Table 3:** Ige sensitization frequency in patients (*n* = 201) and controls (*n* = 201).

Allergen	Asthma patients *n* (%)	Controls *n* (%)	Odds Ratio (lower limit - upper limit, 95% CI)	*p* value
Blo t 2	76 (37.81)	26 (12.94)	3.5 (2.129–5.753)	<0.0001
Blo t 5	106 (52.74)	36 (17.91)	5.12 (3.043–8.606)	<0.0001
Blo t 10	40 (19.90)	12 (5.97)	3.80 (1.893–7.626)	<0.0001
Blo t 12	52 (25.87)	29 (14.43)	2.10 (1.246–3.524)	0.0053
Blo t 13	36 (17.91)	9 (4.48)	4.38 (2.03–9.431)	<0.0001
Blo t 21	109 (54.23)	32 (15.92)	5.05 (3.089–8.265)	<0.0001
Der p 1	88 (43.78)	16 (7.96)	13,00 (5.667–29.824)	<0.0001
Der p 2	99 (49.25)	20 (9.95)	16,80 (6.815–41.414)	<0.0001
Der p 23	95 (47.26)	23 (11.44)	13,00 (5.667–29.824)	<0.0001
ABA-1	33 (16.42)	30 (14.93)	1,13 (0.649–1.95)	0.675
Asc l 5	16 (7.96)	22 (10.95)	0,71 (0.368–1.386)	0.32
*B. tropicalis*	140 (69.65)	42 (20.90)	9,17 (5.052–16.634)	<0.0001
*A. lumbricoides*	114 (56.72)	53 (26.37)	3,54 (2.251–5.571)	<0.0001
*D. pteronyssinus*	127 (63.18)	43 (21.39)	8,64 (4.626–16.123)	<0.0001
Blo t 2/Blo t 5/Blo t 21	138 (68.66)	50 (24.88)	5,89 (3.573–9.706)	<0.0001
Der p 1/Der p 2/Der p 23	127 (63.18)	33 (16.42)	14,43 (6.707–31.04)	<0.0001
*B. tropicalis* + *D. pteronyssinus*	153 (76.12)	63 (31.34)	8,50 (4.673–15.46)	<0.0001
Blo t 2/Blo t 5/Blo t 21/ Der p 1/Der p 2/Der p 23	157 (78.11)	65 (32.34)	6,75 (3.993–11.411)	<0.0001

### 3.2 Diagnostic performance of allergens and extracts

**Figure 3:**
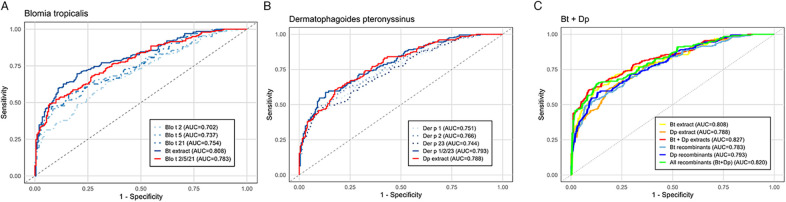
ROC curves of allergens, extracts and combination of allergens. **(A)** *B. tropicalis* (Bt) extract compared to allergens combination. **(B)** *D. pteronyssinus (Dp)* extract compared to allergens combination. **(C)** Two mite extracts vs. two allergen combinations. Asthma diagnosis was the reference standard for ROC analysis.

Random forest analysis indicated that Blo t 10, Blo t 12, Blo t 13, ABA-1 and Asc l 5 contributed minimally to distinguishing asthma patients from controls, whereas Der p 2, Blo t 21, Blo t 5, Der p 1, Der p 23 and Blo t 2 were identified as the most important variables (Supplementary Figure S1). For *B. tropicalis* the area under the curve (AUC) for the combination Blo t 2/Blo t 5/Blo t 21 was similar to that of the extract: 0.783 (95% CI: 0.739–0.827) vs. 0.808 (95% CI: 0.766–0.850) respectively (*p* = 0.421, DeLong's Test). The AUC for individual allergens was 0.702 for Blo t 2 (95% CI: 0.651–0.752), 0.737 for Blo t 5 (95% CI: 0.689–0.785) and 0.754 for Blo t 21 (95% CI: 0.707–0.800) (Figure 3A).

Similarly, for *D. pteronyssinus*, the AUC for the combination Der p 1/Der p 2/Der p 23 was comparable to that of its extract: 0.793 (95% CI: 0.750–0.835) vs. 0.788 (95% CI: 0.745–0.831) (*p* = 0.887). Individual allergens AUCs were 0.751 for Der p 1 (95% CI: 0.705–0.798), 0.766 for Der p 2 (95% CI: 0.721–0.812) and 0.744 for Der p 23 (95% CI: 0.697–0.791) (Figure 3B). Diagnostic performance was highest for the six allergens panel (AUC 0.820 CI: 0.780–0.860) and the combination of both HDM extracts (AUC 0.827, 95% CI: 0.787–0.866) (Figure 3C).

**Figure 4:**
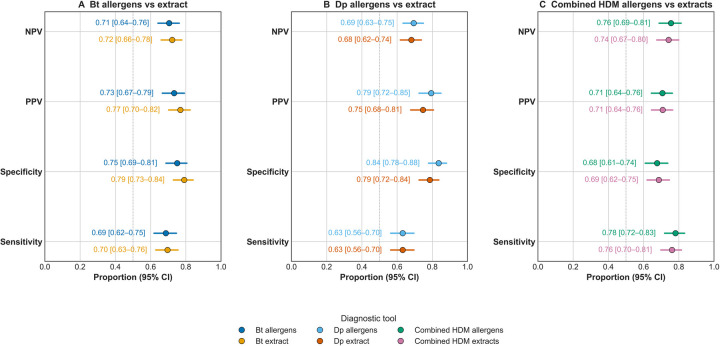
Sensitivity and specificity of specific IgE tests to allergen combinations and extracts. Forest plots show sensitivity, specificity, positive predictive value (PPV), and negative predictive value (NPV), with 95% confidence intervals, for specific IgE tests against (**A**) *Blomia tropicalis* (Bt), (**B**) *Dermatophagoides pteronyssinus* (Dp), and (**C**) the combination of both extracts (HDM), as well as for the corresponding allergen panels: Bt (Blo t 2/5/21), Dp (Der p 1/2/23), and HDM (6 allergens). CI, confidence interval. The dashed vertical line indicates the reference value of 0.5.

Figure 4 shows that *B. tropicalis* extract has similar sensitivity (69.6%) and slightly higher specificity (79.1%) than the Blo t 2/Blo t 5/Blo t 21 allergen combination (68.6% and 75.1%, respectively). For *D. pteronyssinus*, both the extract and the Der p 1/Der p 2/Der p 23 allergen combination had identical sensitivities (63%), but the allergen combination was more specific (83.5%) compared to the extract (78.6%). The use of both extracts had a 76.1% sensitivity and 68.6% specificity, while the six-allergen panel reached a 78.1% sensitivity and 67.6 specificity (Table 4). To examine the level of agreement between the extract and molecular allergens we applied a Kappa analysis and found that most comparisons showed substantial agreement (Table 5).

**Table 4:** Predictive performance of specific IgE against extracts, allergens, and their combinations for diagnosing asthma.

Allergen	Sensitivity, %	Specificity, %	PPV, %	NPV, %
Blo t 2	37.81	87.06	74.51	58.33
Blo t 5	52.74	82.09	74.65	63.46
Blo t 10	19.90	94.03	76.92	54.00
Blo t 12	25.87	85.57	64.20	53.58
Blo t 13	17.91	95.52	80.00	53.78
Blo t 21	54.23	84.08	77.30	64.75
Der p 1	43.78	92.04	84.62	62.08
Der p 2	49.25	90.05	83.19	63.96
Der p 23	47.26	88.56	80.51	62.68
ABA-1	16.42	85.07	52.38	50.44
Asc l 5	7.96	89.05	42.11	49.18
*B. tropicalis*	69.65	79.10	76.92	72.27
*A. lumbricoides*	56.72	73.63	68.26	62.98
*D. pteronyssinus*	63.18	78.61	74.71	68.10
Blo t 2/Blo t 5/Blo t 21	68.66	75.12	73.40	70.56
Der p 1/Der p 2/Der p 23	63.18	83.58	79.38	69.42
*B. tropicalis* + *D. pteronyssinus*	76.12	68.66	70.83	74.19
Blo t 2/Blo t 5/Blo t 21 + Der p 1/Der p 2/Der p 23	78.11	67.66	70.72	75.56

PPV, positive predictive value; NPV, negative predictive value.

**Table 5:** Kappa agreement index, calculated based on the concordance of patients’ sensitization.

Allergen	Kappa	*p*-value	Agreement^a^
Blo t 2/5/21 vs. Bt extract	0.640	<0.0001	**Substantial**
Der p 1/2/23 vs. Dp extract	0.692	<0.0001	**Substantial**
HDM combined vs. extracts combined	0.669	<0.0001	**Substantial**
Blo t 2 vs. Bt extract	0.405	<0.0001	Moderate
Blo t 5 vs. Bt extract	0.621	<0.0001	**Substantial**
Blo t 21 vs. Bt extract	0.555	<0.0001	Moderate
Der p 1 vs. Dp extract	0.613	<0.0001	**Substantial**
Der p 2 vs. Dp extract	0.634	<0.0001	**Substantial**
Der p 23 vs. Dp extract	0.532	<0.0001	Moderate

^a^ Agreement was interpreted according to Cohen's kappa scale: values <0 indicate poor agreement, 0.00–0.20 slight, 0.21–0.40 fair, 0.41–0.60 moderate, 0.61–0.80 substantial, and 0.81–1.00 almost perfect agreement. Bt extract, *B. tropicalis* extract; Dp extract, *D. pteronyssinus* extract. HDM combined=Blo t 2/5/21 + Der p 1/2/23. Extracts combined=*B. tropicalis + D. pteronyssinus* extracts.

### 3.3 Clinical impact of allergens and extracts sensitization

**Figure 5:**
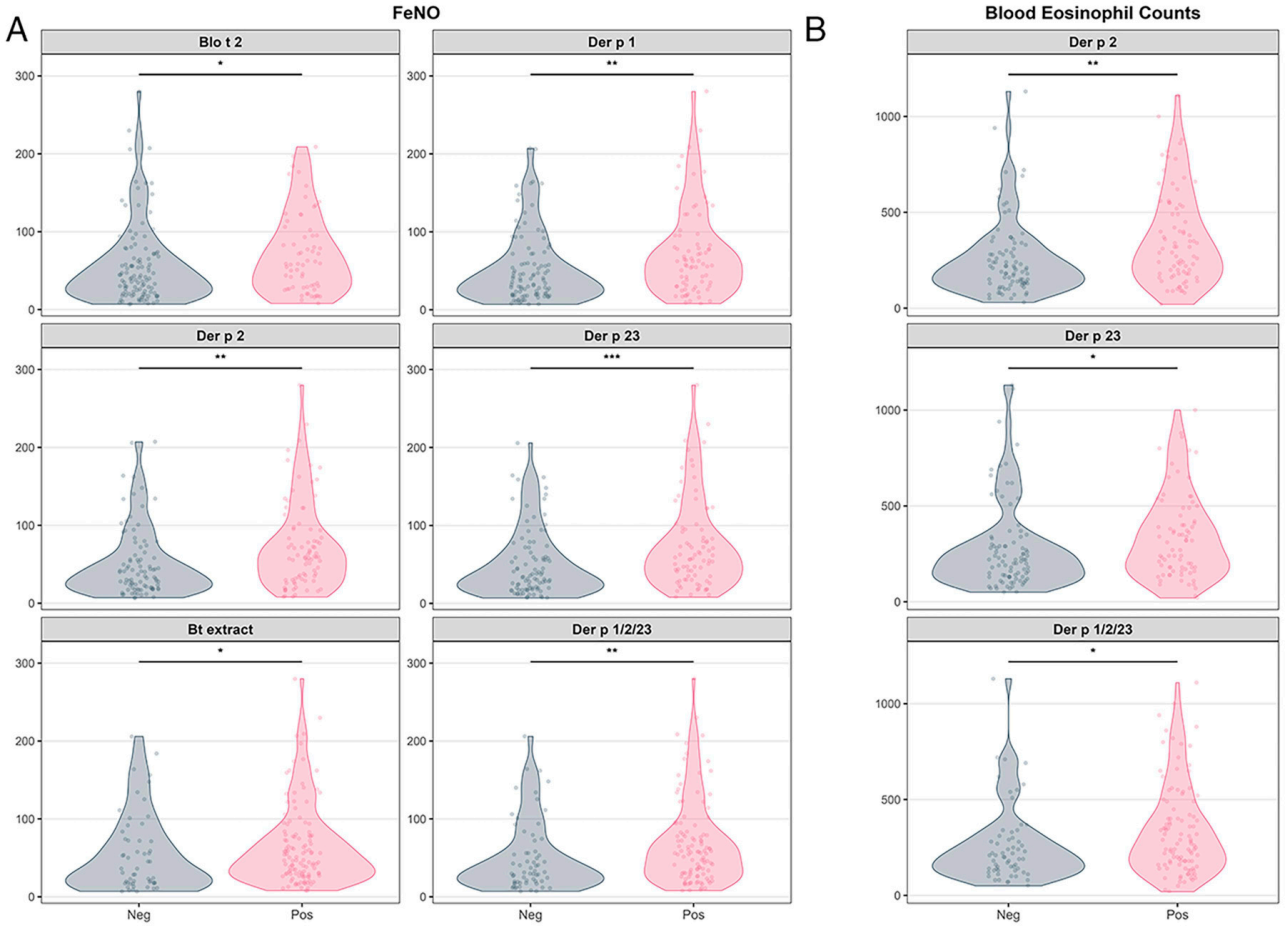
FeNO levels and blood eosinophil counts. **(A)** FeNO levels were higher in asthma patients sensitized to Blo t 2, Der p 1, Der p 2, Der p 23, the combination Der p 1/Der p 2/Der p 23 or *B. tropicalis* extract. **(B)** Differences in eosinophil counts were found in cases sensitized to Der p 2, Der p 23 or Der p1/Der p2/Der p23. *p < 0.05, **p < 0.01, *** *p* < 0.001. Bt, *B. tropicalis* extract.

FeNO levels were significantly higher in asthma patients sensitized to Blo t 2 (*p* = 0.02), Der p 1 (*p* = 0.004), Der p 2 (*p* = 0.002), Der p 23 (*p* = 0.0006), the Der p1/Der p 2/Der p 23 combination (*p* = 0.005), and *B. tropicalis* extract (*p* = 0.048) (Figure 5). Blood eosinophil counts were higher in asthma patients sensitized to Der p 2 (*p* = 0.001), Der p 23 (*p* = 0.02) and the Der p1/Der p 2/Der p 23 combination (*p* = 0.016). In multivariate generalized linear models (adjusted for age and sex) FeNO levels remained significantly associated with sensitization to Blo t 2, Der p 1, Der p 2 and Der p 23, and the Der p1/Der p 2/Der p 23 combination. Sensitization to Der p 23 showed the strongest association (*β* = 0.412; Akaike information criterion AIC = 1,836.9), corresponding to 34%–51% higher FeNO compared with non-sensitized individuals. For eosinophil counts, only Der p 2 sensitization remained significantly associated.

### 3.4 Correlation between skin prick test and specific IgE

Among 178 asthma patients who underwent SPT, the wheal diameter for *D. pteronyssinus* (*n* = 94), correlated significantly with specific IgE to the extract (r = 0.402, *p* = 0.0001), as well as with Der p 1 (r = 0.247, *p* = 0.017), Der p 2 (r = 0.315, *p* = 0.002) and Der p 23 (r = 0.408, *p* = 0.0001). For *B. tropicalis* (*n* = 107), wheal diameter correlated with IgE to the extract (r = 0.374, *p* = 0.0001), Blo t 5 (r = 0.314, *p* = 0.001) and Blo t 21 (r = 0.337, *p* = 0.0001). Significant associations were also found between dichotomous SPT results and specific IgE status for both *B. tropicalis* (*φ* = 0.385, *p* = 0.0001) and *D. pteronyssinus* (*φ* = 0.322, *p* = 0.0001).

## 4 Discussion

**Figure 6:**
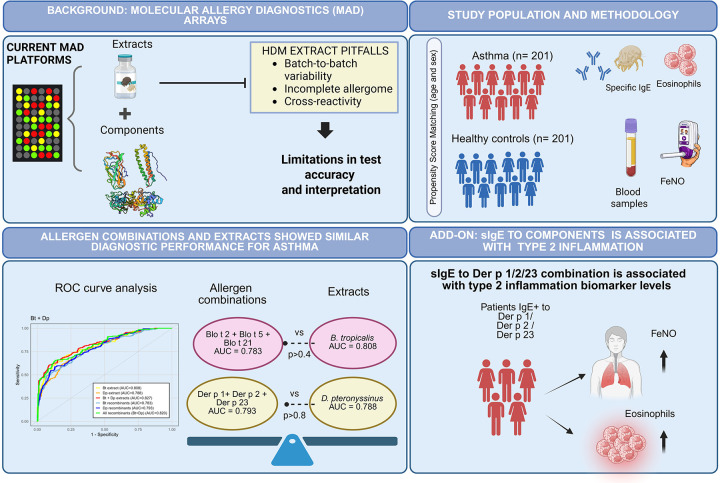
A summary of the rationale, study design and key findings of this study.

This study demonstrates that specific combinations of purified allergens, Blo t 2/Blo t 5/Blo t 21 for *B. tropicalis* and Der p1/Der p 2/Der p 23 for *D. pteronyssinus*, can achieve diagnostic performance equivalent to that of conventional allergen extracts. This conclusion is supported by comparable frequencies of sensitization, AUC values, sensitivity and specificity, and significant associations with asthma biomarkers such as FeNO and blood eosinophil counts. Our findings support the feasibility of transitioning toward extract-free MAD for asthma diagnosis in tropical regions (Figure 6).

Allergic asthma is highly prevalent in the tropics, largely driven by sensitization to HDM (9). Both *D. pteronyssinus* and *B. tropicalis* are common in tropical and subtropical regions, leading to year-round exposure to their allergens (36, 37). Consequently, co-sensitization is frequent and must be accurately diagnosed, as cross-reactivity occurs not only among HDM extracts but also between HDM allergens and IgE-binding molecules from other sources (38). Characterizing asthma phenotype is crucial for effective treatment, and detecting IgE sensitization is essential for a personalized management approach. While skin testing with extracts of *D. pteronyssinus* and *B. tropicalis* is generally sufficient for initiating immunotherapy (2), the current trend is to use MAD for a more precise assessment of sensitization (8), representing an important step toward developing standardized “molecular extracts” for diagnostics and treatment (6).

The HDM allergens evaluated in this study are supported by robust experimental and clinical evidence of their allergenic activity (30, 39). Sensitization to Blo t 2, Blo t 5 and Blo t 21 is highly frequent (18, 19); our results suggest that their combined use captures the majority of clinically relevant epitopes, reducing reliance on extracts. For *D. pteronyssinus*, Der p 1 and Der p 2 are well- established major sensitizers (10–12). The inclusion of Der p 23 is critical, as it has been identified as a major sensitizer across diverse geographic regions, including the tropics (13–17, 30).

Our target combinations also mitigate the risk of cross-reactivity, thereby improving diagnostic specificity. There is no reported cross-reactivity among Der p 1, Der p 2 and Der p 23, or between these molecules and Blo t 5 or Blo t 21. In addition, low cross reactivity between Blo t 2 and Der p 2, as well as low correlation between Blo t 2 and Der p 2 sensitization have been reported (7, 19, 40). While some cross-reactivity has been detected between Blo t 5 and Blo t 21 (18, 41), both allergens have been independently associated with asthma (18), and their inclusion in the panel increases the AUC, suggesting that our population reacts to non-shared epitopes.

Our data show that the defined allergen combinations have AUC values equivalent to their corresponding extracts for distinguishing asthma patients. Notably, the Der p 1/Der p 2/Der p 23 combination was more specific than the *D. pteronyssinus* extract. The observed AUC values indicate moderate diagnostic performance, which must be interpreted in the context of our study design. Unlike previous studies that used positive specific IgE to extracts as the reference standard, we used a clinically confirmed asthma diagnosis, being the diagnostic efficacy of extracts also under evaluation. This approach, though more clinically relevant, introduces greater variability, as asthma is a multifactorial disease where sensitization is only one contributing pathway (42). A key strength of our study is the use of a clinical gold standard, with well-characterized cases and control groups from the same geographical area. Furthermore, we are the first to analyze the relationships between IgE responses to HDM extracts and defined allergen combinations with two important asthma biomarkers.

Notably, HDM extracts showed no association with these biomarkers, suggesting that these critical allergens may be inadequately represented in the extract (43). In contrast, sensitization to individual *D. pteronyssinus* allergens, the allergen combination Der p 1/Der p 2/Der p 23, and Blo t 2, were all associated with elevated FeNO levels, with Der p 23 showing the strongest association. Furthermore, Der p 2 was also uniquely associated with higher blood eosinophil counts, emphasizing its distinct clinical relevance (10). These results suggest that *D. pteronyssinus* allergens may be more strongly associated with the type 2 inflammatory pathophysiology of allergic asthma than those from *B. tropicalis*. While this possibility requires confirmation in future studies, it is supported by experimental evidence: in animal models of allergic asthma, extracts from *B. tropicalis* and *D. pteronyssinus* induce different types of lung inflammation (44). In addition, Der p 23 induces a classic Th2/eosinophilic response (30) whereas Blo t 2 elicits a mixed neutrophilic/eosinophilic pattern (19). Furthermore, a study in children living in a tropical region found higher FeNO levels in children with higher exposure to Der p 1 (45).

Beyond equivalent diagnostic performance and significant association with type 2 biomarkers, our allergen panels offer significant practical advantages in MAD platforms. They provide detailed, species-specific IgE profiles while minimizing diagnostic confusion from cross-reactivity, thereby enhancing analytical robustness and supporting the development of standardized, extract-free diagnostics and tailored immunotherapy. Our findings also lend support to the potential use of these defined allergen combinations in skin prick testing (46).

Our findings are consistent with previous studies using diverse technologies to detect IgE sensitizations to HDM allergen molecules (47, 48), and the components of our proposed combinations are already included in some commercial MAD platforms, providing a practical foundation for the exclusion of *D. pteronyssinus* and *B. tropicalis* extracts. Especially because certain natural *D. pteronyssinus* and *B. tropicalis* extracts lack important allergens showing a considerable variability in composition and content. It has been previously reported that mite extracts showed a 10–60-fold variation regarding the total protein content and, the contents of the major allergens of *D. pteronyssinus* and *B. tropicalis* differed considerably (30–53-fold change) among the extracts. Indeed, Blo t 5 was quantitatively present in <50% of the of the *B. tropicalis* reagents and could not be clearly detected by immunoblotting in the majority of the *B. tropicalis* commercial extracts (49).

This study was conducted in a tropical population, and the potential influence of past *A. lumbricoides* infections (ascariasis) was considered. Although the sensitization to Ascaris extract was more frequent in patients, the strength of the IgE response and sensitization to the specific markers ABA-1 and Asc l 5, did not differ between groups, nor were they associated with asthma biomarkers, suggesting minimal confounding effect.

A potential limitation of this study is the number of allergens evaluated, making it possible that some sensitizations were not detected. However, a previous exploratory analysis using the Allergy Explorer 2 in patients from this dataset, revealed that IgE recognition was most intense for Blo t 5, Blo t 21, Der p 1, Der p 2 and Der p 23, with minimal additional sensitivity to other allergens (50). This and other data from our study suggest that undetected sensitizations likely have negligible diagnostic impact in this population. Indeed, 13% of those positive to B. tropicalis extract were not positive to any of the Blomia recombinant allergens tested, and 20.5% of those positive to the *D. pteronyssinus* extract were not positive to any of the recombinant allergens tested. It must be disclosed that the allergenic extracts used in this study were not deglycosylated and thereby IgE binding to CCD epitopes was plausible. Nevertheless, previous studies suggest that CCD-reactive IgE is not very relevant toward HDM extracts, as has been reported to pollen extracts and other allergenic sources (51). Overall, the use of purified allergen combinations may help to avoid the confounding effect of CCD epitopes in MAD. In addition, our results support previous studies suggesting that allergen extract based tests can be replaced by molecular HDM allergens that are highly reproducible and not dependent on varying qualities of allergen extracts (47).

## 5 Conclusion

In conclusion, this study demonstrates that two defined molecular allergen combinations, each consisting of just three molecules, provide diagnostic performance similar to HDM extracts while showing stronger associations with key asthma biomarkers. These findings support the use of standardized, extract-free allergen panels in molecular allergy diagnostics.

## Data Availability

The original contributions presented in the study are included in the article/Supplementary Material, further inquiries can be directed to the corresponding author.
