# Supplementary material for: Two combinations of house dust mite allergens show similar performance than extracts for asthma diagnosis
Source: Front Allergy. 2026 Apr 10;7:1816013. doi: 10.3389/falgy.2026.1816013 (PMC13106470; doi:10.3389/falgy.2026.1816013)
Supplement: Supplementary file 1 [file Table1.docx]

Supplementary Material

## Supplementary Figure

##
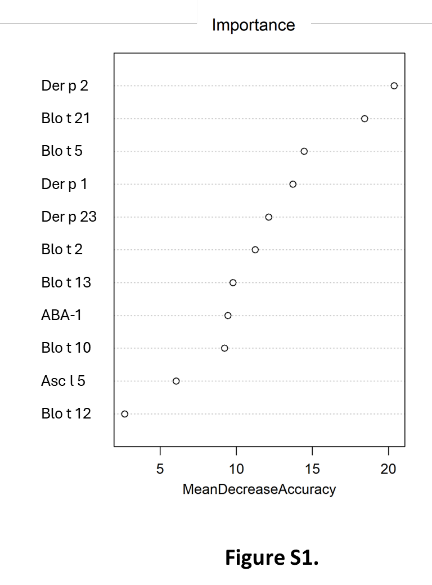


## Supplementary Figure 1. Random forest plot ranking recombinant proteins according to their importance as predictors of asthma, calculated in R using the randomForest package.

**Supplementary Table 1. Sequences of recombinant proteins**

| **Allergen** | **GenBank Access number** | **Protein sequence** |
| --- | --- | --- |
| **Blo t 2** | ABG76185.1 | MFKFICLALL VSYAAAGDVK FTDCAHGEVT SLDLSGCSGD HCTIHKGKSF TLKTFFIANQ DSEKLEIKIS ATMNGIEVPV PGVDKDGCKH TTCPLKKGQK YELDYSLIIP TILPNLKTVT TASLVGDHGV VACGKVNTEV VD |
| **Blo t 5** | AAD10850.1 | MKFAIVLIAC FAASVLAQEH KPKKDDFRNE FDHLLIEQAN HAIEKGEHQL LYLQHQLDEL NENKSKELQE KIIRELDVVC AMIEGAQGAL ERELKRTDLN ILERFNYEEA QTLSKILLKD LKETEQKVKD IQTQ |
| **Blo t 10** | ABU97466.1 | MEAIKKKMQA MKLEKDNAID RAEIAEQKSR DANLRAEKSE EEVRALQKKI QQIENELDQVQESLTQANTK LEEKEKSLQT AEGDVAALNR RIQLIEEDLE RSEERLKVAT AKLEEASHSADESERMRKML EHRSITDEER MDGLESQLKE ARMMAEDADR KYDEVARKLA MVEADLERAEERAETGETKI VELEEELRVV GNNLKSLEVS EEKAQQREEA YEQQIRMMTG KLKEAEARAE FAERSVRKLQ KEVDRLEDEL VHEKEKYKSI SDELDQTFAE LTGY |
| **Blo t 12** | AAA78904.1 | MKSVLIFLVA IALFSANIVS ADEQTTRGRH TEPDDHHEKP TTQCTHEETT STQHHHEEVV TTQTPHHEEK TTTEETHHSD DLIVHEGGKT YHVVCHEEGP IHIQEMCNKY IICSKSGSLW YITVMPCSIG TKFDPISRNC VLDN |
| **Blo t 13** | AAC80579.1 | MPIEGKYKLE KSDNFDKFLD ELGVGFMVKT AAKTLKPTLE VDVQGDTYVF RSLSTFKNTE IKFKLGEEFE EDRADGKRVK TVVNKEGDNK FIQTQYGDKE VKIVRDFQGD DVVVTASVGD VTSVRTYKRI |
| **Blo t 21** | ABH06344.1 | MKFIIALAAL IAVACALPVS NDNFRHEFDH MIVNTATQRF HEIEKFLLHI THEVDDLEKT GNKDEKARLL RELTVSEAFI EGSRGYFQRE LKRTDLDLLE KFNFEAALAT GDLLLKDLKA LQKRVQDSE |
| **Der p 2** | AAF86462.1 | MYKILCLSLL VAAVARDQVD VKDCANHEIK KVLVPGCHGS EPCIIHRGKP FQLEAVFEAN QNTKTAKIEI KASIDGLEVD VPGIDPNACH YMKCPLVKGQ QYDIKYTWNV PKIAPKSENV VVTVKVMGDD GVLACAIATH AKIRD |
| **Der p 23** | XP_027193776.1 | MKFNIIIVFI SLAILVHSSY AANDNDDDPT TTVHPTTTEQ PDDKFECPSR FGYFADPKDP HKFYICSNWE AVHKDCPGNT RWNEDEETCT |
| **ABA-1** | Q06811.2 | GSHHFTLESS LDTHLKWLSQ EQKDELLKMKK DGKAKKELEA KILHYYDELE GDAKKEATEH LKGGCREILK HVVGEEKAAE LKNLKDSGAS KEELKAKVEE ALHAVTDEEK KQYIADFGPA CKKIYGVHTS RRRREFIVTD |
| **Asc l 5** | QGS84239.1 | MKYLITVTCL FVLALVEGQT PSRVPPFLVG APESAVKDFF ELIKKDEEKT DPEIEADIDA FVAKLGADYT NKFIAFKAEL KAHEAEYEKA HAAAIAKFSP AAKEADAKLT AIAEDAKLNG IQKRQKIKET MESLPKEVRD ELEKAIAGGA |

**Supplementary Table 2. Cutoff values for each allergen and extract.**

| **Allergen** | **Cutoff, OD 405nm** |
| --- | --- |
| **Blo t 2** | 0.116 |
| **Blo t 5** | 0.115 |
| **Blo t 10** | 0.111 |
| **Blo t 12** | 0.109 |
| **Blo t 13** | 0.112 |
| **Blo t 21** | 0.102 |
| **Der p 1** | 0.127 |
| **Der p 2** | 0.131 |
| **Der p 23** | 0.124 |
| **ABA-1** | 0.111 |
| **Asc l 5** | 0.119 |
| ***B. tropicalis*** | 0.112 |
| ***A. lumbricoides*** | 0.114 |
| ***D. pteronyssinus*** | 0.122 |

**Supplementary Table 3. Specific IgE levels of cases and controls**

| **Allergen** | **Asthma patients** | | | **Controls** | | **p value** |
| --- | --- | --- | --- | --- | --- | --- |
|  | **n** | **median (IQR)** | **n** | | **median (IQR)** |  |
| **Blo t 2** | 76 | 0.160 (0.132 - 0.223) | 26 | | 0.127 (0.117 – 0.141) | **<0.0001** |
| **Blo t 5** | 106 | 0.303 (0.141 – 0.710) | 36 | | 0.129 (0.119 – 0.172) | **<0.0001** |
| **Blo t 10** | 40 | 0.152 (0.117 -0.640) | 12 | | 0.114 (0.113 – 0.130) | **0.002** |
| **Blo t 12** | 52 | 0.134 (0.115 – 0.189) | 29 | | 0.114 (0.113 - 0.134) | **0.005** |
| **Blo t 13** | 39 | 0.148 (0.121 – 0.197) | 9 | | 0.117 (0.115 – 0.128) | **0.006** |
| **Blo t 21** | 109 | 0.237 (0.132 - 0.399) | 32 | | 0.108 (0.105 – 0.273) | **<0.0001** |
| **Der p 1** | 88 | 0.479 (0.226 – 0.990) | 16 | | 0.189 (0.156 – 0.358) | **0.009** |
| **Der p 2** | 99 | 0.972 (0.386 – 1.765) | 20 | | 0.264 (0.149 – 0.747) | **0.002** |
| **Der p 23** | 95 | 0.360 (0.190 – 0.575) | 23 | | 0.188 (0.135 – 0.307) | **0.001** |
| **ABA-1** | 33 | 0.115 (0.115 – 0.132) | 30 | | 0.122 (0.115 – 0.132) | **0.032** |
| **Asc l 5** | 16 | 0.126 (0.122 – 0.132) | 22 | | 0.128 (0.122 – 0.136) | 0.442 |
| ***B. tropicalis*** | 140 | 0.261 (0.147 – 0.491) | 42 | | 0.137 (0.119 – 0.179) | **<0.0001** |
| ***A. lumbricoides*** | 114 | 0.145 (0.124 – 0.188) | 53 | | 0.132 (0.119 – 0.155) | **0.023** |
| ***D. pteronyssinus*** | 127 | 0.238 (0.150 – 0.479) | 43 | | 0.145 (0.132 – 0.180) | **<0.0001** |
